# 5-Aminolevulinic Acid as a Novel Therapeutic for Inflammatory Bowel Disease

**DOI:** 10.3390/biomedicines9050578

**Published:** 2021-05-20

**Authors:** Vipul Yadav, Yang Mai, Laura E. McCoubrey, Yasufumi Wada, Motoyasu Tomioka, Satofumi Kawata, Shrikant Charde, Abdul W. Basit

**Affiliations:** 1 Intract Pharma Limited, London Bioscience Innovation Centre, London NW1 0NH, UK; 2 School of Pharmaceutical Sciences (Shenzen), Sun Yat-sen University, Guangzhou 510275, China; maiy6@mail.sysu.edu.cn; 3 Department of Pharmaceutics, UCL School of Pharmacy, University College, London WC1N 1AX, UK; laura.mccoubrey.18@ucl.ac.uk; 4 Neopharma Japan, Iidabashi Grand Bloom 4th Floor, 2-10-2 Fujimi, Chiyoda-ku, Tokyo 102-0071, Japan; Yasufumi.Wada@cslbehring.com (Y.W.); tommy@neopharmajp.com (M.T.); satofumi.kawata@neopharmajp.com (S.K.); scharde@gmail.com (S.C.)

**Keywords:** ulcerative colitis, Crohn’s disease, inflammation, 5 amino levulinic acid, colonic drug delivery, anti-inflammatory, large intestine, drug stability, microbiota metabolism, microbiome

## Abstract

5-Aminolevulinic acid (5-ALA) is a naturally occurring nonprotein amino acid licensed as an optical imaging agent for the treatment of gliomas. In recent years, 5-ALA has been shown to possess anti-inflammatory and immunoregulatory properties through upregulation of heme oxygenase-1 via enhancement of porphyrin, indicating that it may be beneficial for the treatment of inflammatory conditions. This study systematically examines 5-ALA for use in inflammatory bowel disease (IBD). Firstly, the ex vivo colonic stability and permeability of 5-ALA was assessed using human and mouse fluid and tissue. Secondly, the in vivo efficacy of 5-ALA, in the presence of sodium ferrous citrate, was investigated via the oral and intracolonic route in an acute DSS colitis mouse model of IBD. Results showed that 5-ALA was stable in mouse and human colon fluid, as well as in colon tissue. 5-ALA showed more tissue restricted pharmacokinetics when exposed to human colonic tissue. In vivo dosing demonstrated significantly improved colonic inflammation, increased local heme oxygenase-1 levels, and decreased concentrations of proinflammatory cytokines TNF-α, IL-6, and IL-1β in both plasma and colonic tissue. These effects were superior to that measured concurrently with established anti-inflammatory treatments, ciclosporin and 5-aminosalicylic acid (mesalazine). As such, 5-ALA represents a promising addition to the IBD armamentarium, with potential for targeted colonic delivery.

## 1. Introduction

The global prevalence of inflammatory bowel disease (IBD) has risen significantly in the last 30 years, with an increase of 85.1% in global cases from 1990 to 2017 [1,2]. IBD carries a heavy burden for both patients and healthcare providers [3]. In the U.S, lifetime healthcare costs for individuals with IBD average at over half a million dollars [4]. Physiologically, patients suffer from noninfectious chronic inflammation of the gastrointestinal (GI) tract, resulting in common symptoms of abdominal pain, diarrhoea, weight loss, rectal bleeding, and malnutrition [5,6]. It is recognised that these symptoms significantly impact patients’ wellbeing and life chances: people with IBD statistically require more days off work than the general population [7,8]. The umbrella term IBD can be distinguished into two clinically distinct conditions: Crohn’s disease (CD) and ulcerative colitis (UC). In CD, inflammation can occur at any point along the GI tract and is typically transmural, affecting all layers of intestinal tissue [9,10]. In juxtaposition, GI inflammation in UC is confined to the colon and only affects mucosal tissue. The precise aetiology of both CD and UC remains incompletely characterised, though existing treatments mostly target a defective immune response [11,12,13].

Existing IBD treatments do not adequately control disease in all patients. Initial treatments, including mesalamine (5-aminosalicylic acid, 5-ASA) corticosteroids, ciclosporin, tacrolimus and azathioprine, often do not lead to disease remission, and carry the risk of significant adverse effects [14,15,16,17,18,19,20,21,22]. Patients who do not respond to first line therapies are typically offered biologic treatment, such as anti-TNF monoclonal antibodies (mAbs) like infliximab, adalimumab, or golimumab [12,13,23,24,25]. To date, there are no orally administrable biologic therapies to treat IBD, thus patients must attend regular healthcare appointments to receive treatment [26]. The antibodies also act as systemic immunosuppressants, leading to multiple adverse effects, including an increased risk of lymphoma [27]. Small molecule based JAK inhibitors for treatment of moderate to severe UC also carry their risks, currently marked as ‘black box’ treatments due to post-marketing reports of pulmonary embolism [28,29]. Even though the emergence of IL12/23 and 23 inhibiting mAbs have been shown to overcome some of the safety challenges of anti-TNF mAbs, the risk of surgery for UC and CD patients is still high, with a substantial proportion of patients needing surgical intervention during their lifetimes [30,31,32]. Clearly, there remains a need for safe, efficacious and early interventional IBD treatments that prevent intestinal damage, and thus disease worsening and surgeries [33]. 

**Figure 1:**
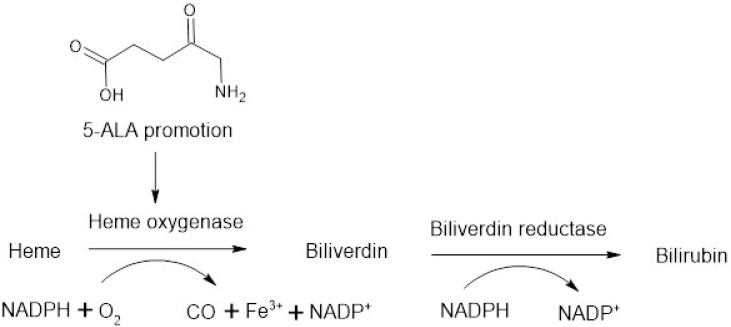
Catabolism of heme, liberating carbon monoxide, iron, biliverdin, and bilirubin. The enzyme heme oxygenase-1 is induced by 5-ALA [44].

5-Aminolevulinic acid (5-ALA) is a naturally occurring nonprotein amino acid. Endogenously, 5-ALA plays a key role in both heme production and catabolism [1,2]. Heme is an essential component of the haemoglobin tetramer, and also contributes towards healthy mitochondrial function [34]. Over the last 30 years, there has been much attention on the therapeutic potential of 5-ALA [35]. The small molecule has been cited as showing promise for treatment of numerous diseases, including type 2 diabetes mellitus, endometriosis, and neurodegeneration [36,37,38]. 5-ALA has also recently been shown to inhibit SARS-CoV-2 infection in vitro [39]. Despite the wide scientific applications, 5-ALA is only licensed by the U.S. Food and Drug Administration for use as an optical imaging agent in patients with high-grade gliomas [40]. Administered orally, 5-ALA acts as a prodrug, whereby it is converted to fluorescent protoporphyrin (PpIX) by tumour cells, thus facilitating fluorescence-guided surgical resection [35]. Subsequently, 5-ALA has significantly increased patients’ progression free survival and the amount of tumour removable during surgery, with a good safety profile [41]. Interestingly, during clinical trials, researchers noticed that 5-ALA was associated with false-positive fluorescence in up to 35% of tumour biopsies [35,42]. This fluorescent activity in the absence of tumour cells was attributed partly to 5-ALA uptake by non-cancerous inflammatory cells [43]. Uptake of 5-ALA in inflammatory conditions could have significant benefits for treatment of IBD. Following 5-ALA promoted heme oxygenase-1 (HO-1) catalysed heme catabolism, carbon monoxide, iron, biliverdin, and bilirubin are liberated (Figure 1) [44]. Together, these molecules are thought to have antioxidative functions and regulate inflammation, apoptosis, fibrosis, angiogenesis, and cell proliferation through distinct yet synergistic effects [2]. Though the additive mechanisms are not yet fully characterised, their clinical relevance has been demonstrated in various disease states, from protection in renal ischaemia reperfusion injury, immunoregulation in heart transplantation, and amelioration of cardiomyocyte hypertrophy [45,46,47]. The delivery of 5-ALA to the colon has also been documented, specifically for photodynamic detection of colon cancer and colitis [48,49]. HO-1, the rate-limiting enzyme in heme catabolism, has been shown to play a protective role in intestinal damage [50,51,52]. Thus, HO-1 induction by 5-ALA may present a novel addition to the existing arsenal of IBD therapeutics.

This study seeks to investigate the suitability of 5-ALA for treatment of IBD. Firstly, the ex vivo stability of 5-ALA in the colon and its colonic permeability are investigated using human and mouse tissue. Secondly, the efficacy of 5-ALA, in the presence of sodium ferrous citrate (SFC), is determined using an acute DSS colitis mouse model of IBD. SFC is formulated with 5-ALA as an iron component, to support 5-ALA’s ability to promote HO-1 formation (Figure 1). Both oral and intracolonic routes are investigated, and 5-ALA performance is directly compared to that of two traditional IBD treatments: 5-ASA (mesalamine) and ciclosporin.

## 2. Materials and Methods

### 2.1. Materials

5-ALA hydrochloride, 5-ASA, haemin, L-cysteine HCl, vitamin K, and resazurin were obtained from Sigma Aldrich, Gillingham, UK. SFC was provided by Neopharma Japan. Ciclosporin was obtained from Cayman Chemical, Ann Arbor, Michigan, USA. Trifluoroacetic acid, tween 80, and sodium bicarbonate were obtained from Sigma Aldrich, UK. Sodium chloride and dipotassium hydrogen phosphate were obtained from Fisher Chemical, Loughborough, UK. Magnesium sulphate heptahydrate and calcium chloride hexahydrate were obtained from VWR, Poole, UK. Bile salts were from Fluka Analytical, Buchs, Switzerland. Boric acid, phosphate buffer saline, Nonidet P-40, and fluorescamine were obtained from Sigma Aldrich, UK. DSS was obtained from MP Biomedicals, Santa Ana, CA, USA. A protease fluorescent detection kit was purchased from Sigma-Aldrich, MI, USA. All other chemicals used were of high-performance liquid chromatography (HPLC)-grade and were used as received.

### 2.2. Methods

#### 2.2.1. 5-ALA Stability and Permeability in Colonic Conditions

##### Preparation of Colonic Fluid

All faecal work was conducted within an anaerobic workstation (Electrotek 500TG workstation, Electrotek, West Yorkshire, UK) set at 37 °C and 70% relative humidity. Faeces from healthy human volunteers were weighed inside the chamber to total 5 g. Each 5 g sample was then combined with 20 g basal medium (Table 1), homogenised at 10,000 rpm, and sieved through a mesh with 5 µm aperture to remove any solid material. The resulting faecal slurries from 3 volunteers were pooled at equal ratios to increase microbiota diversity within slurry. Pooled faecal slurries were used immediately after preparation. The same method was also used to prepare a separate slurry produced from mouse faeces (C57BL6 healthy mice, *n* = 10) to provide validation of 5-ALA stability in the murine colon during following in vivo work. 

##### Stability of 5-ALA in Faecal Slurry (Colonic Fluid)

The stability of 5-ALA was assessed through incubation with faecal slurry for 24 h in an anaerobic chamber at 37 °C and 70% relative humidity. A 12 mg/mL solution of 5-ALA in HPLC-grade water was added to a 2 mg/mL solution of SFC in HPLC-grade water; these were then combined with faecal slurry in a 1:1 ratio. During incubation, samples were agitated at 100 rpm on a horizontal shaker (VXR basic Vibrax^®^, Leicestershire, UK). Aliquots of the reaction mixture were removed for analysis at timepoints (hours): 0, 1, 2, 4, 6, and 24. Withdrawn aliquots for each timepoint were immediately combined with ice cold quencher solution (0.5% trifluoroacetic acid) at a ratio of 1:2 to halt microbiota activity in slurry. Samples were then centrifuged at 9600× *g* for 10 min at 4 °C. The supernatant was collected and analysed using HPLC with fluorescence (HPLC-FLD). As a negative control, 5-ALA was incubated in basal medium only, in the absence of faecal microbiota. The reaction and control were both conducted in triplicate. 

##### HPLC-FLD Quantification of 5-ALA

A method by Namjoshi et al. for the derivatisation of 5-ALA to a fluorescent moiety was used to detect 5-ALA [53]. In short, a 0.1% fluorescamine solution was made by dissolving 10 mg fluorescamine in 10 mL acetone. A borate buffer was produced by dissolving 61.83 mg boric acid in 10 mL HPLC water and adjusting the pH to 8.5 with 5 M sodium hydroxide. Subsequently, 100 µL of the fluorescamine solution and 300 µL of borate buffer were added to 100 µL of 5-ALA sample (removed from the incubation experiment) and vortexed. The resulting solution was rested at room temperature for 10 min. Next, this solution was analysed using HPLC-FLD system (1260 Infinity II Series™, Agilent Technologies, Didcot, UK) equipped with a pump (model G1311C), autosampler (model G1329B), and a diode-array UV detector (model G1314B). Analysis was performed at room temperature with a fluorescence detection wavelength of 395/480 nm excitation/emission. An injection volume of 10 µL for each 5-ALA sample was pumped through a 150 × 4.6-mm Jupiter 5 μm 300 Å (Phenomenex, Torrance, CA, USA) C18 column at 1.0 mL/min. An isocratic mobile phase consisting of 70% water and 30% acetonitrile was used. The area of the dominant chromatograph peak was used to quantify 5-ALA, using two polynomial regression models developed using triplicate standards of derivatised 5-ALA in mouse faecal slurries.

##### Preparation of Colonic Tissue Lysate for Assessment of 5-ALA Stability

Human colonic tissue was supplied as a surgical byproduct during routine colonic resection from Royal Free Hospital (*n* = 1). Murine colonic tissue (strain C57BL6, *n* = 3) was collected postmortem after culling mice in CO_2_ and immediate surgical removal. Tissue samples were transported in ice and used fresh. Tissue homogenate was prepared using an extraction buffer (Table 2).

Colonic tissue was weighed, and 100 mg was added to freshly prepared extraction buffer, producing a mixture equaling 5 mL. This mixture was then homogenised at 10,000 rpm for 20 s and incubated for 2 h at 4 °C to allow complete extraction of tissue enzymes. Next, the mixture was centrifuged at 10,000 rpm for 10 min at 4 °C. The resultant supernatant containing tissue enzymes was then assessed for protease activity using the protease fluorescent detection kit. Each tissue enzyme sample produced was added to a solution of fluorescein isothiocyanate-casein (FITC-casein) and incubated for 1 h at 37 °C under shaking (70 rpm). Trichloroacetic acid (0.6 M) was then added to the mixture and incubated for a further 30 min under the same conditions. The resultant mixture was centrifuged at 10,000× *g* for 10 min at room temperature, supernatant collected, and analysed spectrophotometrically at 485/535 nm excitation/emission. The assumption was that one unit of protease within samples would hydrolyse FITC-casein and produce a fluorescence intensity equivalent to 1 ng of trypsin FITC-casein per hour at 37 °C. Proteases within samples were quantified accordingly. 

##### Stability Assessment of 5-ALA in the Presence of Colonic Tissue Enzymes 

Colonic tissue enzyme stability studies were conducted within an anaerobic chamber (Electrotek 500TG workstation, Electrotek, West Yorkshire, England) set at 37 °C and 70% relative humidity. A 120 mg/mL 5-ALA in HPLC water solution was added to a 20 mg/mL SFC solution, and together mixed with colonic tissue enzyme supernatant at a ratio of 1:19 (final concentration 5-ALA: 6 mg/mL, SFC: 1 mg/mL). This mixture was then incubated, and aliquots removed for analysis at timepoints (hours): 0, 1, 2, 4, 6, and 24. At each timepoint, the withdrawn aliquot was added to ice cold quencher solution (0.5% trifluoroacetic acid) at a ratio of 1:2, to halt all enzymatic action. Samples were then centrifuged at 9600× *g* for 10 min at 4 °C. The supernatant was collected and processed according to the 5-ALA derivatisation and HPLC-FLD method. All incubations were conducted in triplicate, and incubation of 5-ALA in extraction buffer in the absence of colonic tissue enzymes was used as a negative control.

##### Evaluation of 5-ALA Colonic Tissue Permeability

Both human and murine colonic tissue was obtained as above. Excised tissue was used fresh and transferred as soon as possible to ice cold Krebs–Ringer bicarbonate (Kbr) buffer of pH 7.4 to maintain viability. Mouse colonic tissue was cut open transversally and washed with Kbr buffer to remove luminal contents. A NaviCyte vertical Ussing chamber system (Harvard Apparatus, Cambridge, UK) was used to measure 5-ALA permeability across the colonic tissue. The Ussing chamber system was prepared so that colonic tissue (size: 4 × 8 mm^2^, exposed tissue area: 0.28 cm^2^) was mounted between two chambers containing 4 mL Kbr buffer on each side, with the apical side facing one and the basolateral side facing the other. The drug was added on the apical side to measure the transcytosis across the mucosa into the tissue and across to the basal side. Epithelial tissue integrity was assured using an EVOM™ voltammeter (World Precision Instruments Inc., Hertfordshire, UK) to measure the transepithelial electrical resistance (TEER) of the tissue. A TEER value of 200 Ω/cm^2^ was set as the lower limit to confirm tissue viability and tight junction integrity. Ussing chambers were purged with carbogen (95% O_2_, 5% CO_2_) and maintained at 37 °C by water jackets throughout experimentation. Before addition of 5-ALA, tissue mounted between the Ussing chambers was incubated in the Kbr buffer with monitoring of TEER. Then, 5-ALA and SFC were added to the apical side at concentrations of 6 mg/mL and 1 mg/mL, respectively. The permeation of 5-ALA was tested over 3 h in triplicate. A negative control, whereby no drug was added to the apical chamber, was used. Quantification of 5-ALA in the basolateral Ussing chamber (signalling epithelial permeation) was conducted using the HPLC-FLD method as above.

#### 2.2.2. Efficacy in DSS Colitis Mouse Model

##### Animals and Study Design

A combination of 5-ALA + SFC at two doses (10 + 1.5 mg/kg and 100 + 15.7 mg/kg) were tested for efficacy in a dextran sulfate sodium (DSS) mouse model of colitis. These doses were chosen as part of the dose finding strategy, as 5-ALA has not previously been tested in in vivo models of IBD. The standard first doses during preclinical testing of new IBD therapeutics are in the range of 10 mg/kg and 100 mg/kg [54]. Doses were administered both orally (PO) by gavage and intrarectally (IR), and compared against two established treatments for DSS colitis (positive controls): ciclosporin and 5-ASA. Male C57BL/6 mice (Jackson Laboratories) were initiated into the study from 9 weeks old. All mice were housed in HEPA-ventilated static rate cages (IPC, Innovive, San Diego, CA, USA) with sani-chips bedding. Fluorescent lighting was provided on a 12-h cycle. Temperature and humidity were monitored daily and maintained to the best extent possible between 20 and 23 °C, and 30–70% humidity. This in vivo study took place at Crown Bioscience laboratories (San Diego, CA, USA) and respected animal welfare in line with the U.S. Department of Agriculture’s Animal Welfare Act (9 CFR Parts 1, 2, 3). All processes followed company standard operating procedures and were conducted solely by qualified personnel under the management of a study director. 

**Figure 2:**
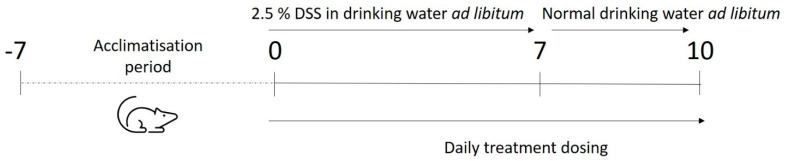
Overview of the study design. Mice were acclimatised for 7 days, then commenced group-specific treatment with concurrent colitis induction via DSS. All mice were sacrificed on day 10.

An overview of the study design can be seen in Figure 2, with details on dosing for treatment groups in Table 3 Animals were sorted randomly into 11 groups (*n* = 7 per group) and allowed to acclimatise to their surroundings for 7 days. Following this, normal drinking water was replaced with 2.5% DSS in drinking water, with ad libitum consumption, to induce colitis similar to that seen in IBD. At the same time, daily dosing of the treatment commenced and continued for a total of 10 days (Table 3). After 7 days of treatment, DSS consumption in drinking water ceased and normal water was resumed. The PO and IR naïve mice groups 1 and 7 were not administered DSS at any time during the study. All animals were sacrificed on day 10.

5-ALA, SFC, and 5-ASA were formulated by suspension in phosphate buffered saline (PBS) at specified concentrations and vortexed briefly until dissolved. Ciclosporin was formulated in olive oil and ethanol at a ratio of 9:1. Animals were dosed according to their body weight and group allocation.

##### Clinical Observations

Body weight, stool consistency, and stool blood content were measured daily for each animal for the duration of the study.

- Weight score: 0 (maintain or increase in weight), 1 (1–5% weight loss), 2 (6–10% weight loss), 3 (11–15% weight loss), 4 (>16% weight loss).
- Stool consistency score: 0 (normal), 1 (moist/sticky), 2 (soft), 3 (diarrhoea).
- Stool blood content score: 0 (negative haemoccult test—no blood), 1 (positive haemoccult test in >30 s), 2 (positive haemoccult test in <30 s), 3 (gross observable blood).

For humane reasons, animals were sacrificed prematurely if they showed sustained weight loss of ≥20% for up to 72 h (as per U.S. animal welfare regulations). Disease activity index (DAI) was calculated as a representation of all clinical observations, whereby DAI equaled the sum of body weight, stool consistency, and stool blood content scores. 

##### Postmortem Analysis of Plasma and Tissue

Following sacrifice, terminal blood plasma was collected from mice of all groups by cardiac puncture (>0.2 mL) and placed into heparinised plasma separator tubes (Becton, Dickinson and Company, Franklin Lakes, NJ, USA). Plasma was collected after centrifugation, aliquoted into 50 µL portions, and stored at −80 °C. During autopsy, animals’ spleens were removed and weighed. Colon length and weight was measured, then two 2 cm sections of colon (furthest proximal and distal sections) were collected for each animal, and flash frozen in liquid nitrogen. Plasma and colonic tissue were analysed for the following chemokines/cytokines: TNF-α, IL-1β, IL-6, IL-10, and IL-2 using a Luminex^®^ multianalyte profiling kit (Invitrogen, Carlsbad, CA, USA). Plasma and colonic tissue HO-1 was also quantified using an ELISA assay (Abcam Mouse SimpleStep ELISA, Cambridge, UK).

#### 2.2.3. Data Analysis and Statistics

Both Python (version 3.9.0, via Jupyter Notebook version 6.1.4) and Microsoft Excel (via Office 365) were used to store, analyse, and plot data. Statistical significance between measurements’ means was determined using one-way analysis of variance (ANOVA). A *p*-value of <0.05 was deemed significant.

## 3. Results and Discussion

### 3.1. Stability and Permeability of 5-ALA in Colonic Conditions

**Figure 3:**
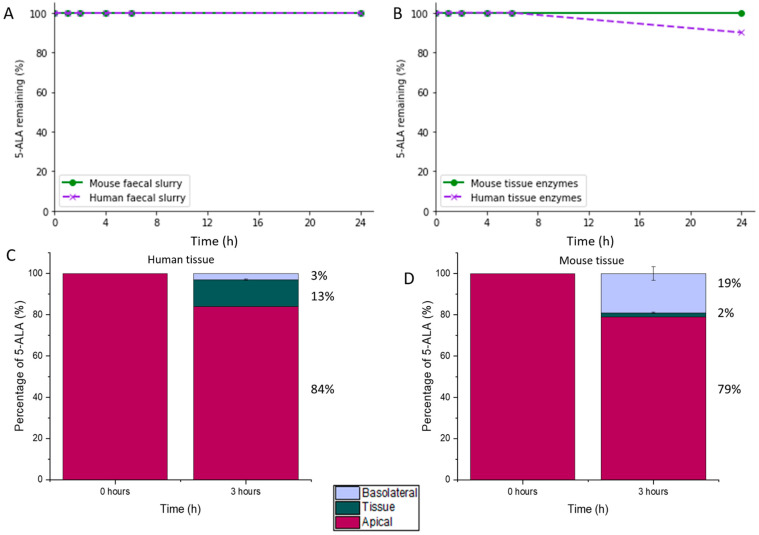
Stability profile of 5-ALA during, (**A**): incubation with faecal slurry (human (*n* = 3) and mouse (*n* = 10)); (**B**): incubation with colonic tissue enzymes (human (*n* = 1) and mouse (*n* = 3)); (**C**): percentage of 5-ALA in different compartments during Ussing experiment with human colonic tissue (1 donor); (**D**): percentage of 5-ALA in different compartments during Ussing experiment with mouse colonic tissue (*n* = 3). Permeation of 5-ALA occurs from the apical to basolateral side of tissue.

5-ALA was found to be completely stable in both mouse and human faecal slurry with no drug degradation observed over 24 h (Figure 3A). As the total intestinal transit time of the general human population is around 27 h, it can be assumed that 5-ALA will not be microbially degraded in the GI tract [55].

Favourably, 5-ALA was seen to maintain stability in the presence of human and mouse colonic tissue enzymes (Figure 3B). During incubation with human colonic tissue enzymes, a 10% degradation was observed between 6 and 24 h. In mice, total stability was maintained throughout the 24 h. During tissue characterisation, human colonic tissue was found to have a pH of 7.01, and protease activity of 61,256 (±8123) units/mL. Mouse tissue had a slightly lower pH of 6.71 and protease activity of 54,782 (±8394) units/mL. Enzymes in the intestinal wall can contribute towards drug degradation [56], thus it is important to characterise drug susceptibility before oral administration to properly predict drug fate in situ. From this data, degradation of 5-ALA by colonic tissue enzymes is not expected to significantly affect drug pharmacokinetics.

The colonic permeability study found that 3% of available 5-ALA was able to permeate across human colon epithelial tissue within 3 h. In addition, 13% of available 5-ALA was found within the colonic tissue, mid-permeation (Figure 3C). Using mouse tissue, 19% of 5-ALA diffused across the colonic epithelium fully after 3 h, and 2% was in the process of tissue permeation (Figure 3D). This data provides a basis for the assumption that 5-ALA delivered to the colonic lumen will largely remain gut-restricted, extending its availability for local therapeutic action.

### 3.2. Efficacy in an IBD Mouse Model

#### 3.2.1. Disease Activity Index (DAI) Score

**Figure 4:**
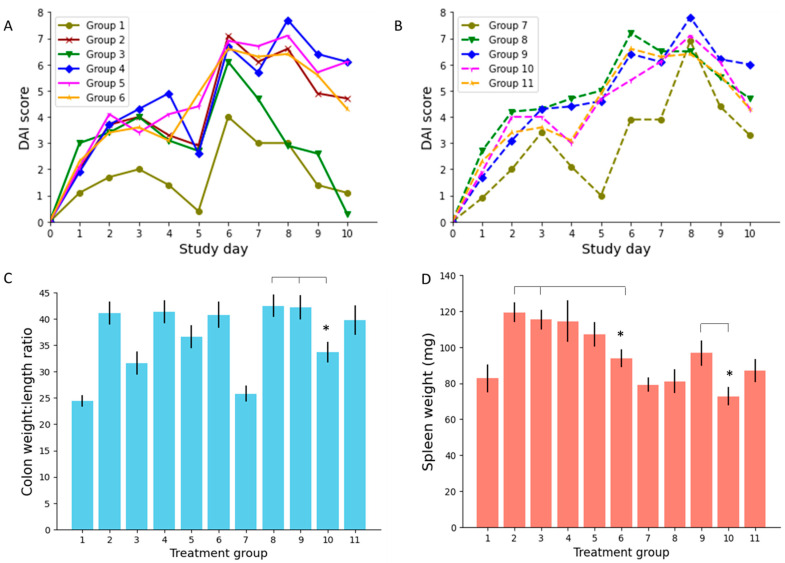
(**A**): Daily mean disease activity index (DAI) scores for PO treatment groups; (**B**): DAI scores for IR treatment groups; (**C**): mean colonic weight to length ratios per treatment group; (**D**): mean spleen weights per treatment group. *n* = 7 mice per treatment group. Error bars: standard deviation. The * marker indicates significant 5-ALA superiority where *p* < 0.05.

The DAI scores from days 0–10 for mice in each treatment group of the study are presented in Figure 4A,B. Low dose PO 5-ALA (group 5) did not record statistically lower DAI scores than other PO treatments during the study. High dose PO 5-ALA (group 6), however, achieved significantly lower DAI scores compared to PO 5-ASA (group 4) on day 10 (*p* = 0.008). Both low and high dose IR 5-ALA treatments recorded lower DAI scores than the IR 5-ASA treatment (group 9) on day 10; however, these results did not reach statistical significance.

#### 3.2.2. Colon Weight to Length Ratio

Low dose 5-ALA administered intrarectally (group 10) was seen to significantly lower colonic weight to length ratio compared to IR ciclosporin (group 8, *p* = 0.01) and IR 5-ASA (group 9, *p* = 0.02) (Figure 4C). Similarly, PO low dose 5-ALA (group 5) achieved lower ratios than PO 5-ASA (group 4), though this did not reach statistical significance (*p* = 0.15). Colitis is known to increase the colonic weight to length ratio [57]. Accordingly, effective treatments for colonic inflammation should reduce this ratio to mirror that measured in healthy colons.

#### 3.2.3. Spleen Weight

Average spleen weights for animals in this study can be seen in Figure 4D. No statistical significance was observed between the spleen weights of animals administered with PO ciclosporin, PO 5-ASA, or PO low dose 5-ALA (groups 3–5, *p* = 0.18). However, PO high dose 5-ALA (group 6) did significantly reduce spleen weight compared to untreated colitis (group 2, *p* = 0.03) and PO ciclosporin (group 3, *p* = 0.01). This suggests that PO 5-ALA has notably improved intestinal inflammation [58], demonstrated by its significance over untreated controls, and has achieved this to an acceptable therapeutic extent, exhibited by its significance over PO ciclosporin. Similarly, both dose levels of IR 5-ALA (groups 10 and 11) accomplished significantly lower spleen weights than untreated controls (group 2, *p* = 0.002 and 0.01, respectively). Treatment with low dose IR 5-ALA (group 10) achieved spleen weights lower than IR 5-ASA (group 9, *p* = 0.02).

#### 3.2.4. Colonic Tissue and Serum Analysis

##### Plasma Inflammatory Markers

**Figure 5:**
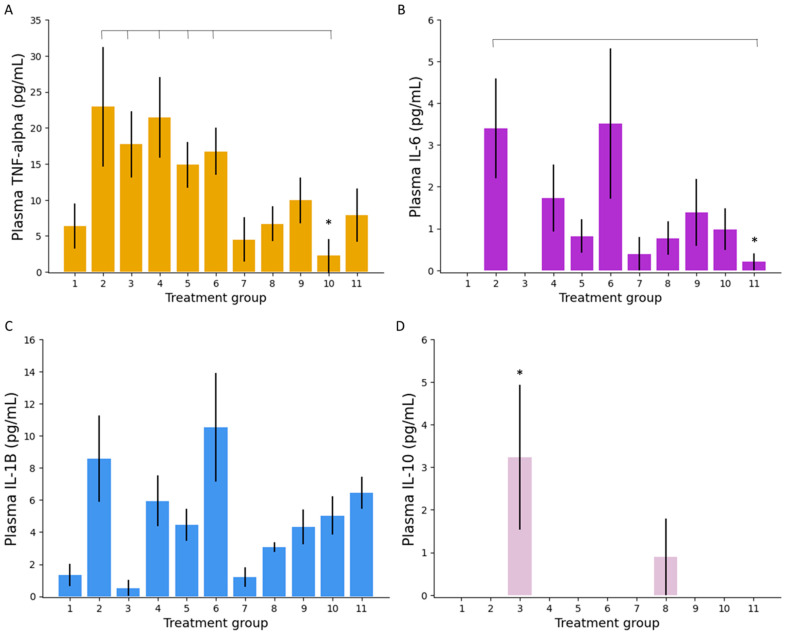
Plasma concentrations of inflammatory markers: TNF-α (**A**); IL-6 (**B**); IL-1β (**C**); and the anti-inflammatory marker, IL-10 (**D**), in mice following 10 days of treatment. *n* = 7 mice per treatment group. Error bars: SEM, the * marker indicates significant 5-ALA/SFC superiority where *p* < 0.05.

Analysis of inflammatory markers in mouse serum and colonic tissue was conducted to gain an appreciation of treatments’ effect on local and systemic inflammation. Plasma TNF-α, as shown in Figure 5A, was found to be significantly lower in mice treated with low dose IR 5-ALA (group 10) compared to untreated colitis (group 2, *p* = 0.03), PO ciclosporin (group 3, *p* = 0.009), PO 5-ASA (group 4, *p* = 0.007), and, interestingly, both PO 5-ALA groups (groups 5 and 6, *p* = 0.005 and 0.002, respectively). Low dose IR 5-ALA (group 10) also achieved lower plasma TNF-α levels compared to IR 5-ASA (group 9) and IR ciclosporin (group 8), though neither of these reached significance (*p* = 0.05 and 0.16, respectively). This indication that colitis was superiorly treated by IR 5-ALA is echoed by the plasma concentrations of IL-6, as shown in Figure 5B. Group 11 (high dose IR 5-ALA) achieved significantly lower IL-6 plasma concentrations than untreated controls (group 2, *p* = 0.02), not seen with any other treatments. Groups 1 (PO PBS control) and 3 (PO ciclosporin) had plasma IL-6 concentrations that were below the limit of detection. 5-ALA treatments did not significantly lower inflammatory plasma concentrations of IL-1β compared to other treatments (Figure 5C). However, both doses of IR 5-ALA resulted in a lower average plasma IL-1β concentration than untreated controls (group 2). Measurement of plasma IL-10 does, however, suggest that PO ciclosporin (group 3) was able to initiate anti-inflammatory pathways better than any other treatment (*p* = 0.077); in fact, no other groups achieved significantly measurable plasma IL-10 levels (Figure 5D). No treatments were shown to modulate the levels of IL-2 (data not shown).

##### Colonic Tissue Inflammatory Markers

**Figure 6:**
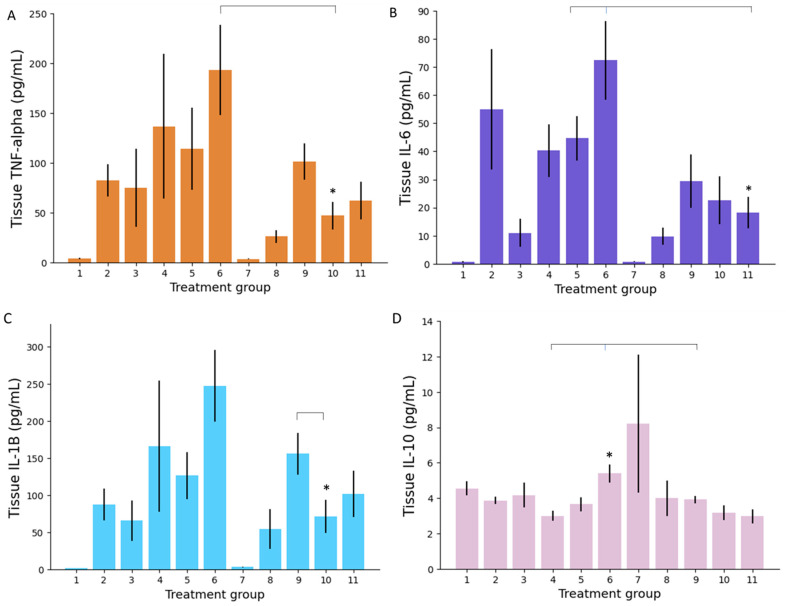
Colonic tissue concentrations of inflammatory markers: TNF-α (**A**); IL-6 (**B**); and IL-1β (**C**); and the anti-inflammatory marker, IL-10 (**D**), in mice following 10 days of treatment. *n* = 7 per treatment group. Error bars: SEM, the * marker indicates significant 5-ALA/SFC superiority where *p* < 0.05.

Concentrations of the inflammatory markers in animals’ colonic tissue were different, though correlative, to those noted in plasma. Firstly, IR 5-ALA once again recorded lower TNF-α concentrations than when administered orally (Figure 6A). Low dose IR 5-ALA (group 10) performed the best of the 5-ALA therapies, achieving significantly lower tissue TNF-α concentrations than high dose PO 5-ALA (group 6, *p* = 0.01). There was no significant difference between low dose IR 5-ALA (group 10) and other IR treatment groups, including the group with the lowest mean TNF-α concentration (group 8, IR ciclosporin, *p* = 0.18). Colonic tissue IL-6 concentrations again showed group 11 (high dose IR 5-ALA) to be the best performing 5-ALA therapy (Figure 6B). Group 11 achieved significantly lower IL-6 colonic tissue concentrations than PO low dose 5-ALA (group 5, *p* = 0.02) and PO high dose 5-ALA (group 6, *p* = 0.004). There was no significant difference between the lowest IL-6 treatment average (group 8, IR ciclosporin) and groups 10 and 11 (IR 5-ALA), indicating that IR 5-ALA was just as effective as a potent immunosuppressive agent like ciclosporin. This was reaffirmed by IL-1β levels, wherein IR 5-ALA formulations were statistically comparative to ciclosporin formulations (Figure 6C). Low dose IR 5-ALA (group 10) also achieved statistically lower IL-1β concentrations than IR 5-ASA (group 9, *p* = 0.04). Treatment with high dose IR 5-ALA (group 11) achieved similar IL-1β results to the low dose IR 5-ALA formulation (group 10); however, it did not result in significantly better results than IR 5-ASA (group 9). Reduction in proinflammatory markers TNF-α, IL-6 and IL-1β demonstrates the significant superiority of IR 5-ALA treatment compared to PO treatments in the reduction of inflammatory cytokines in colonic tissue. 

With regards to the anti-inflammatory marker IL-10, concentrations were substantially higher in all treatment groups within colonic tissue compared to plasma (*p* = 2.67 × 10^−25^). The drug treatment group achieving the highest mean tissue concentration of IL-10 was high dose PO 5-ALA (group 6), which was statistically greater than PO 5-ASA (group 4, *p* = 0.001) and IR 5-ASA (group 9, *p* = 0.03) (Figure 6D). Although IL-2 concentrations were significantly higher in colonic tissue compared to plasma, there was no significant difference between study groups (data not shown). 

Herein, 5-ALA has shown the ability to suppress inflammatory pathways both systemically and locally. When comparing the different methods of 5-ALA administration, the IR route generally demonstrated enhanced anti-inflammatory activity compared to the PO route. Furthermore, there does not seem to be a cumulative dose–activity relationship; on several occasions, administration of low dose IR 5-ALA (group 10) has resulted in lower concentrations of inflammatory markers than high dose IR 5-ALA (group 11). This is surprising, as animals in group 11 received a tenfold dose increase compared to those in group 10. These results could indicate that efficacy of 5-ALA is dose-saturable, with dose increases above a certain threshold having no appreciating benefit.

##### Heme Oxygenase Activity

5-ALA is known to induce the upregulation of HO-1, an enzyme involved in protection against intestinal IBD damage (Figure 1) [31,32,33]. 

**Figure 7:**
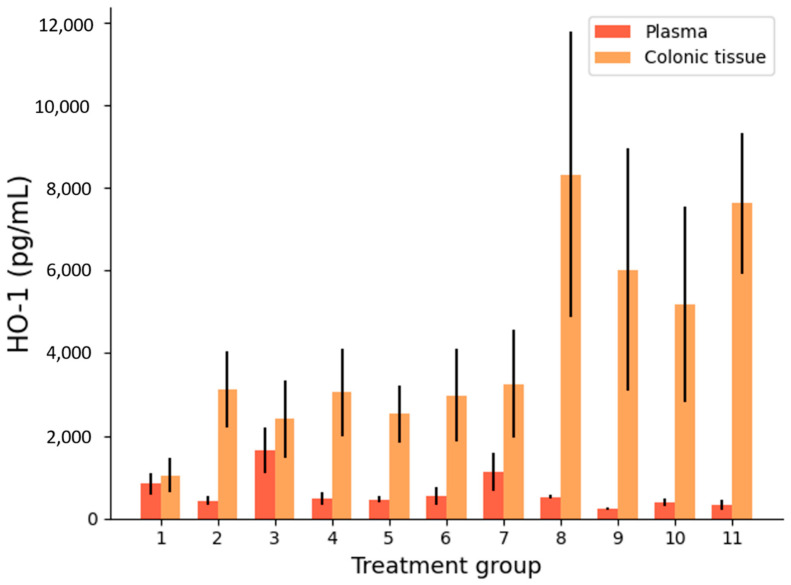
Heme oxygenase-1 (HO-1) concentrations in plasma and colonic tissue per group, after 10 days of treatment. *n* = 7 mice per treatment group. Error bars: SD.

Figure 7 shows that HO-1 concentrations tended to be much higher in colonic tissue than plasma in all treated and untreated DSS arms (*p* = 8.12 × 10^−5^ across all groups). High HO-1 levels in tissue compared to plasma are a positive sign that there is a local anti-inflammatory response to colitis. This is likely partially explained by an intrinsic physiological response to intestinal inflammation, as group 1 (the colitis-free control) had similar levels of HO-1 in plasma and colonic tissue, as opposed to group 2 (the untreated colitis control) with significantly more HO-1 in colonic tissue than plasma (*p* = 1.23 × 10^−5^). In addition, an increased colonic tissue HO-1 concentration can also be attributed to localised drug delivery, as shown by the IR formulations (groups 8–11). Groups 8, 9, and 11 achieved significantly higher concentrations of colonic tissue HO-1 than the IR placebo (group 7), showing that drug treatment does increase tissue HO-1 (*p* < 0.05). Of the IR treatments, only group 10 (low dose 5-ALA) did not show statistical superiority over the IR placebo (group 7). In general, IR treatments resulted in higher HO-1 colonic tissue concentrations than the PO treatments (*p* = 1.72 × 10^−7^). This is an indication that local drug delivery has greater ability to exert local drug action. High dose IR 5-ALA (group 11) induced colonic tissue HO-1 activity to a greater extent than all PO treatments (*p* < 0.0005).

### 3.3. The Potential of 5-ALA as a Novel IBD Treatment

The results of this study have presented 5-ALA as a promising addition to the IBD armamentarium. Firstly, 5-ALA is stable in the colonic environment, exhibiting resistance to degradation by colonic microbiota and tissue enzymes. Many drugs are susceptible to chemical transformation by colonic microbial enzymes, thus this data implies that 5-ALA can be relied upon to maintain a predictable concentration profile at its site of action [59,60,61]. In addition, 5-ALA did not show extensive permeability across the human colonic epithelium, indicating that it will primarily remain restricted in the colon tissue for local action where it is needed, with minimal systemic exposure and any associated adverse effects. When tested in a mouse model of colitis, 5-ALA demonstrated efficacy across numerous therapeutic indicators, including spleen weight, colon weight: length ratio, local and systemic inflammatory cytokines, and local HO-1 concentrations. 

No clear adverse effects of 5-ALA treatment were observed during this preclinical study. In line with drug development guidelines, 5-ALA’s toxicity profile will need to be systematically assessed for treatment of IBD; though, when used as an imaging agent, 5-ALA is recognised as a safe molecule [41,62]. In the case of SFC, iron supplements have a well-documented safety profile. Overall, 5-ALA delivered intrarectally achieved better results than when administered orally. Local delivery of drugs at the site of disease is often an efficacious way to enhance therapeutic action whilst decreasing the incidence of systemic side effects and the required dose [63]. Though IR administration of 5-ALA has been shown to be superior to PO administration in this study, a promising hybrid of the two could come in the form of targeted colonic delivery via the oral route [64]. Oral formulations that release the drug in the colon encompass the therapeutic advantages seen with local IR efficacy, whilst offering the patient acceptability and convenience seen with PO formulations [65,66]. 5-ALA could be delivered directly to the colon using targeted formulation. For example, Phloral^®^ technology, used commercially for delivery of a high-dose mesalazine product for treatment of ulcerative colitis, could be applied to colonic 5-ALA delivery. Phloral^®^ allows drugs to be orally administered and reliably delivered to the colon by releasing drug in response to shifts in intestinal pH and microbial enzymatic activity [67,68,69]. Finally, another interesting finding of this study was the lack of linear dose–response relationship observed for 5-ALA. Two doses of 5-ALA were tested: 10 mg/kg and a tenfold increase of 100 mg/kg. In numerous instances, low dose 5-ALA outperformed the high dose group, for example, in plasma/tissue TNF-α and IL-1β concentrations. That said, in some cases, the converse was true; for example, high dose IR 5-ALA achieved significant results over all PO treatments in tissue HO-1 concentration, where the low dose IR group did not. Future dose-ascending studies should be employed to investigate 5-ALA’s relationship with dose and efficacy in IBD.

## 4. Conclusions

This study assessed the viability and efficacy of 5-ALA as a novel first-line treatment for IBD. Results demonstrated that 5-ALA is stable in the presence of colonic microbiota and tissue enzymes for up to 24 h. In addition, 5-ALA was revealed as being largely gut-restricted, with low concentrations able to diffuse across the human colonic epithelium. When tested in an IBD mouse model, 5-ALA was shown to decrease plasma and colonic tissue concentrations of inflammatory cytokines TNF-α, IL-6, and IL-1β. Such immunomodulatory effects were frequently superior to that seen with established IBD treatments, ciclosporin and 5-ASA. The anti-inflammatory enzyme, HO-1, was also significantly upregulated locally in colonic tissue by 5-ALA treatment. As delivery of 5-ALA was shown to achieve greater therapeutic benefit than traditional oral administration, the therapy would be ideally suited for oral targeted colonic delivery. Here, 5-ALA has demonstrated promise as a novel addition to the arsenal of therapeutics aimed at early interventional treatment of IBD. Future studies should focus on investigating 5-ALA’s dose–efficacy relationship and explore its specific delivery to the colon via the oral route. 

## 5. Patents

Intract Pharma Limited and Neopharma Japan have filed a patent for the therapeutic use of compositions containing 5-aminolevulinic acid for the local treatment of inflammatory bowel disease. International Publication Number: WO 2020/221827 A1.

## Figures and Tables

**Table 1:** Composition of the basal medium used to support anaerobic microbiota growth in faecal slurry. Components were dissolved in HPLC-grade water sequentially as listed, under stirring. Basal medium was left to stabilise for 30 min prior to use.

Component	Quantity/L (In Grams Unless Otherwise Stated)
Peptone water	2.00
Yeast extract	2.00
Sodium chloride	0.10
Monopotassium phosphate	0.04
Calcium chloride hexahydrate	0.01
Magnesium sulfate heptahydrate	0.01
Tween 80	2 mL
Bile salts	0.50
L-cysteine hydrochloride	0.50
Phytomenadione (vitamin K)	10 µL
Sodium bicarbonate	2.00

**Table 2:** Composition of the extraction buffer used to extract colonic tissue proteins. Components were added under stirring and allowed to stir until complete dissolution of sodium chloride.

Component	Quantity per 10 mL HPLC H_2_O
Tris-buffered saline (10X stock solution)	2 mL
Phosphate buffered saline	8 mL
Sodium chloride	146 mg
Nonidet TM P40 substitute	100 µL

**Table 3:** Treatment group description.

Group	Number of Mice	Route	Intervention	Therapeutic Dose (mg/kg)
1	7	PO	PBS	NA
2	7	PO	2.5% DSS + PBS	NA
3	7	PO	2.5% DSS + ciclosporin	70
4	7	PO	2.5% DSS + 5-ASA	50
5	7	PO	2.5% DSS + 5-ALA/SFC	10/1.5
6	7	PO	2.5% DSS + 5-ALA/SFC	100/15.7
7	7	IR	PBS	NA
8	7	IR	2.5% DSS + ciclosporin	70
9	7	IR	2.5% DSS + 5-ASA	50
10	7	IR	2.5% DSS + 5-ALA/SFC	10/1.5
11	7	IR	2.5% DSS + 5-ALA/SFC	100/15.7

## Data Availability

Not applicable.
